# Conservative Management for Left Atrial Intramural Hematoma After Cryoballoon Ablation

**DOI:** 10.1016/j.jaccas.2025.106328

**Published:** 2025-12-03

**Authors:** Taku Shimojo, Naoaki Onishi, Ren Kimura, Kiyotaka Shimamura, Yohei Kobayashi, Tomohisa Tada, Chinatsu Yamada, Haruyasu Ito, Fujio Hayashi

**Affiliations:** a Department of Cardiology, Japanese Red Cross Osaka Hospital, Osaka, Japan; b Department of Cardiology, Hyogo Prefectural Amagasaki General Medical Center, Hyogo, Japan

**Keywords:** atrial fibrillation, conservative management, cryoballoon ablation, echocardiography, large-bore sheath, left atrial intramural hematoma

## Abstract

**Background:**

Left atrial intramural hematoma (LAIH) is an extremely rare complication associated with left atrial procedures.

**Case Summary:**

We report the case of a 50-year-old man who developed LAIH during cryoballoon ablation for paroxysmal atrial fibrillation. The lesion was detected immediately after the procedure by routine intracardiac echocardiography, enabling prompt management. Because the patient remained hemodynamically stable, conservative management with bed rest and close monitoring was chosen. The hematoma showed gradual regression, and the patient was discharged on postoperative day 13 without secondary complications.

**Discussion:**

Although surgical intervention is often required in hemodynamically unstable patients, this case demonstrated that conservative management can be an effective strategy for hemodynamically stable patients with LAIH.

**Take-Home Messages:**

LAIH can be successfully managed with conservative treatment in hemodynamically stable patients. Early recognition and timely intervention are essential for complete resolution.

## Visual Summary

**Figure:**
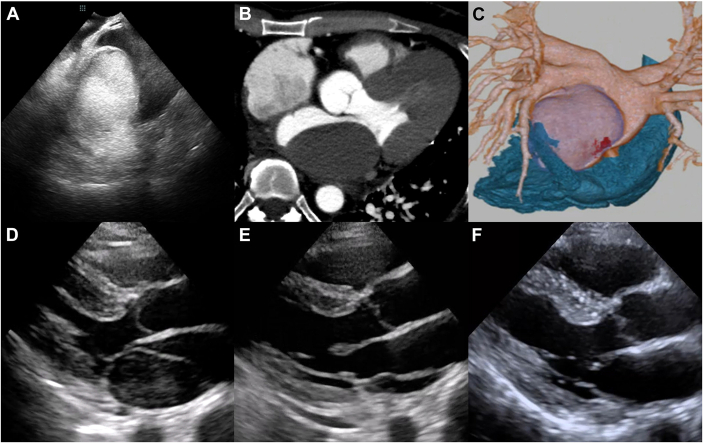

**Visual Summary:**
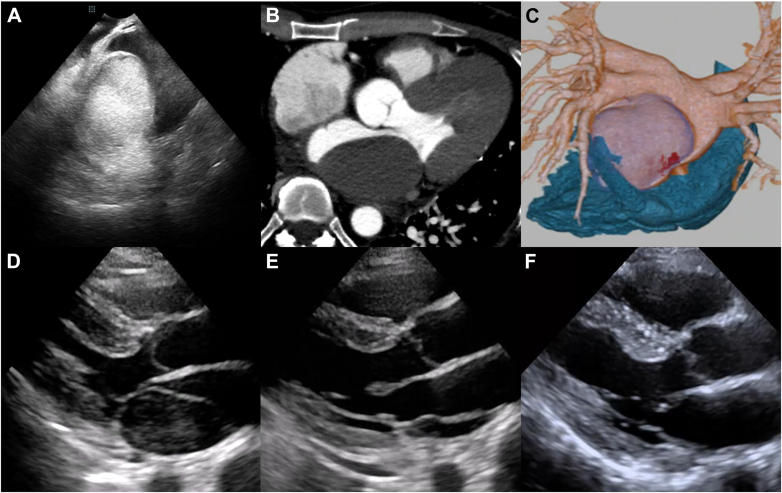
Multimodal Imaging and Temporal Change of Left Atrial Intramural Hematoma With Progressive Resolution (A) Intracardiac echocardiography imaging of LAIH. (B and C) Axial imaging of cardiac CT and 3-dimensional reconstruction of cardiac CT posterior view, showing the spatial relationship of the right heart, left atrium, pulmonary veins, and LAIH. (D to F) TTE images of the LAIH on postoperative day 1, postoperative day 4, and 4 months after discharge, respectively. CT = computed tomography; LAIH = left atrial intramural hematoma; TTE = transthoracic echocardiography.

## History of Presentation

**Figure 1:**
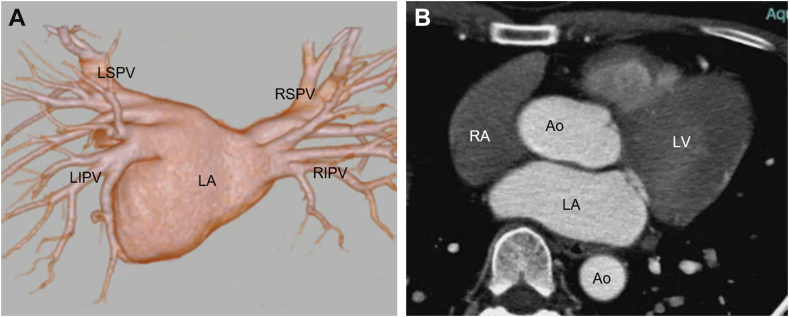
Cardiac CT of the Left Atrium and Pulmonary Veins (A) Posterior view showing normal anatomy of the left atrium and pulmonary veins. (B) The left atrium is not dilated. Ao = aorta; LA = left atrium; LIPV = left inferior pulmonary vein; LSPV = left superior pulmonary vein; LV = left ventricle; RA = right atrium; RIPV = right inferior pulmonary vein; RSPV = right superior pulmonary vein.

A 50-year-old man with symptomatic paroxysmal atrial fibrillation (AF) was referred to our department for catheter ablation owing to recurrent palpitations refractory to antiarrhythmic drug therapy. Transthoracic echocardiography (TTE) showed normal left ventricular systolic function (ejection fraction: 66%) and no left atrial enlargement (left atrial diameter: 36 mm). Cardiac computed tomography (CT) demonstrated normal pulmonary venous anatomy (Figures 1A and 1B). Transesophageal echocardiography performed 1 day before the procedure revealed no evidence of left atrial appendage thrombus or pericardial effusion.

**Figure 2:**
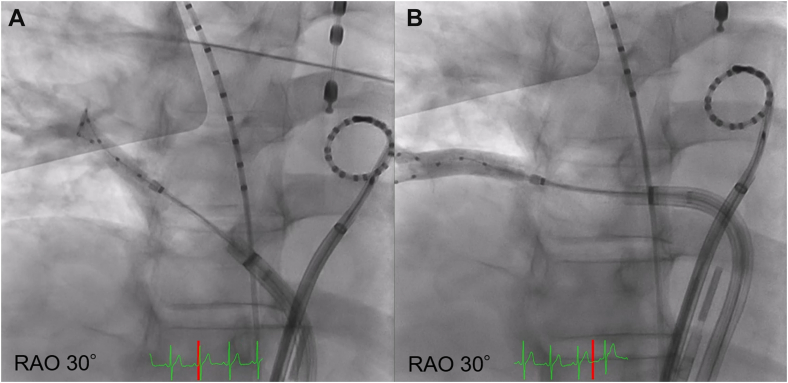
Cannulation to the Right Inferior Pulmonary Vein With a Cryoballoon (A) Cannulation to the superior branch and the (B) inferior branch of the RIPV. RAO = right anterior oblique; RIPV = right inferior pulmonary vein.

**Figure 3:**
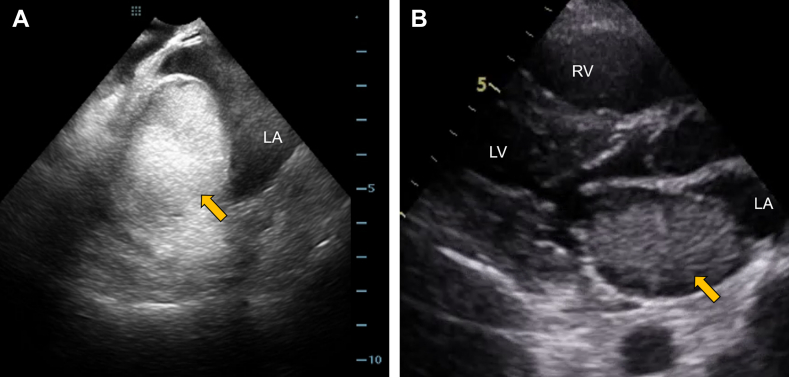
Echocardiographic Images of the Left Atrial Intramural Hematoma (A) Intracardiac echocardiography showed a high echoic structure in the left atrium (arrow). (B) TTE showed a mass occupying most of the left atrium cavity (arrow). LA = left atrium; LAIH = left atrial intramural hematoma; LV = left ventricle; RV = right ventricle; TTE = transthoracic echocardiography.

Pulmonary vein isolation was performed using cryoballoon ablation. On the day of the procedure, the patient was in sinus rhythm. The ablation was performed under local anesthesia with conscious sedation using propofol. Bilateral femoral venous access was obtained, and intravenous heparin was administered to maintain an activated clotting time of approximately 300 to 350 seconds. Transseptal access to the left atrium was achieved via a single puncture using a Brockenbrough needle (Scooper RFN, Fukuda Denshi) under both fluoroscopic and intracardiac echocardiographic guidance. Two transseptal sheaths (FlexCath Advance, Medtronic, and SL0 8.5-F, Abbott Medical Japan) were introduced into the left atrium. A cryoballoon catheter (Arctic Front Advance Pro, Medtronic) and a circular mapping catheter (Inquiry Optima, Abbott Medical Japan) were advanced into the pulmonary veins. The pulmonary veins were isolated in the following order: left superior pulmonary vein, left inferior pulmonary vein, right inferior pulmonary vein (RIPV), and right superior pulmonary vein. The left superior pulmonary vein, left inferior pulmonary vein, and right superior pulmonary vein were successfully isolated with a single freeze of 150 to 160 seconds per vein. However, the isolation of the RIPV was technically challenging. Initially, a 20-mm circular mapping catheter (Achieve Advance, Medtronic) was advanced into the superior branch, but isolation was unsuccessful because of suboptimal occlusion of the cryoballoon (Figure 2A). The sheath was then deflected to redirect the mapping catheter into the inferior branch, which improved balloon apposition, enabled cooling to −50 °C, and resulted in successful isolation (Figure 2B). Bidirectional conduction block was confirmed in all pulmonary veins after ablation. During this process, insertion of the circular mapping catheter into the RIPV was technically difficult. Intracardiac echocardiography performed at the end of the procedure showed no pericardial effusion; however, an approximately 5-cm intracardiac mass was revealed within the posterior wall of the left atrium (Figure 3A).

## Past Medical History

The patient's medical history was unremarkable. At the time of referral, his medications included flecainide 50 mg twice daily and rivaroxaban 15 mg daily. Flecainide was discontinued 1 day before the procedure, and rivaroxaban was withheld on the day of the procedure.

## Differential Diagnosis

The differential diagnosis included left atrial intramural hematoma (LAIH), left atrial thrombosis, and pericardial effusion with tamponade.

## Investigations

**Figure 4:**
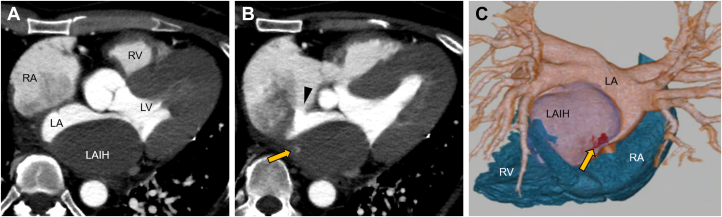
Cardiac CT of the Left Atrial Intramural Hematoma (A) Axis imaging of the LAIH. (B) Contrast extravasation into the LAIH (arrow). The black arrowhead indicates the iatrogenic atrial septal defect. (C) Three-dimensional reconstruction of cardiac CT posterior view showing the spatial relationship of the right heart, left atrium, pulmonary veins, and LAIH. The red portion (arrow) indicates contrast extravasation. CT = computed tomography; LA = left atrium; LAIH = left atrial intramural hematoma; LV = left ventricle; RA = right atrium; RV = right ventricle.

Intracardiac echocardiography suggested the presence of an intramural hematoma in the left atrial wall (Figure 3A). Protamine was administered immediately to reverse the effects of heparin. TTE demonstrated a highly echogenic structure occupying the most of the left-atrial cavity, consistent with an expanding intramural hematoma (Figure 3B). No pericardial effusion was observed. The mitral valve mean pressure gradient was 3 mm Hg, and the tricuspid regurgitation pressure gradient measured 22 mm Hg, indicating the absence of mitral stenosis and pulmonary hypertension. Cardiac CT confirmed the diagnosis of LAIH, measuring approximately 60 × 35 mm on axial imaging (Figure 4A). Contrast extravasation into the hematoma cavity was detected in the inferomedial aspect of the lesion, suggesting active bleeding (Figures 4B and 4C). No pulmonary vein stenosis or obstruction was identified.

## Management

**Figure 5:**
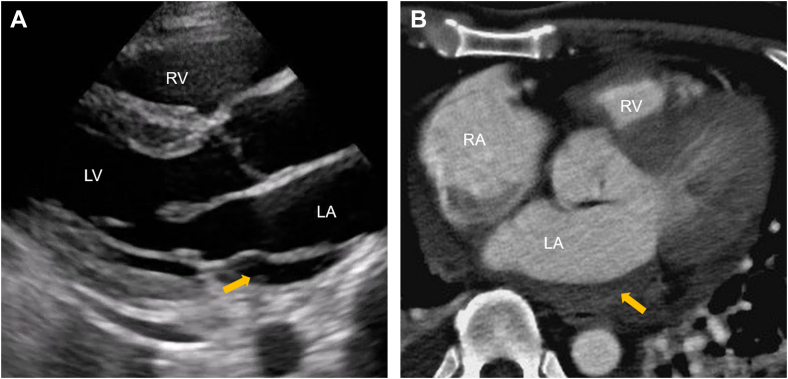
Transthoracic Echocardiography and Cardiac CT on Postoperative Day 4 (A and B) TTE and cardiac CT showed a sudden decrease in the size of the LAIH (arrow). TTE = transthoracic echocardiography; other abbreviations as in Figure 4.

**Figure 6:**
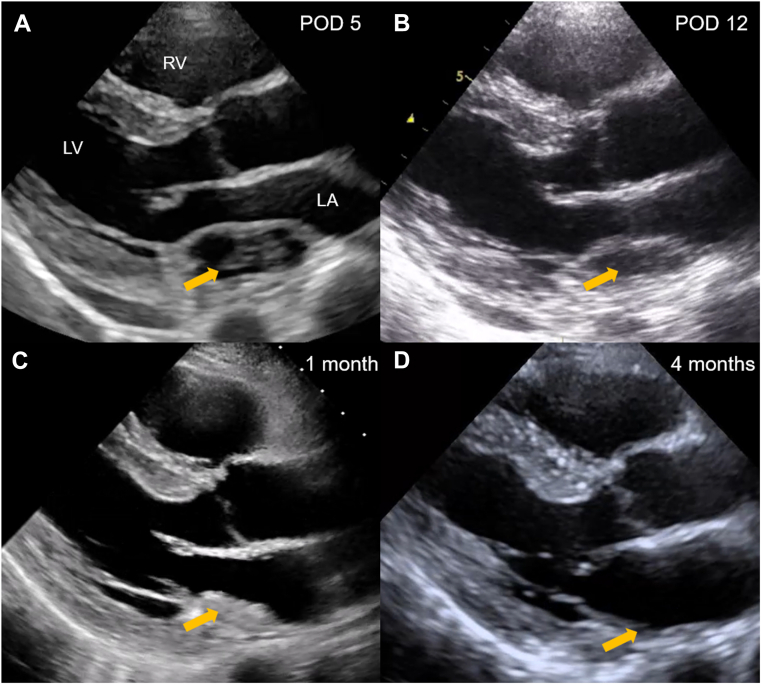
Serial Transthoracic Echocardiography Images TTE performed on (A) postoperative day 5 and (B) postoperative day 12. A slight re-expansion occurred on postoperative day 5, subsequently demonstrating continued gradual regression in hematoma size (arrow). TTE images at (C) 1 month and (D) 4 months after discharge. LAIH demonstrated continued gradual regression, with near-complete resolution observed 4 months after discharge (arrow). POD = postoperative day; TTE = transthoracic echocardiography; other abbreviations as in Figure 4.

Although the patient's vital signs remained stable, he was admitted to the intensive care unit for close monitoring and was placed on strict bed rest. Surgical repair was considered; however, a conservative management strategy was selected because of the patient's hemodynamic stability and the absence of clinical deterioration. On the following day, repeat cardiac CT demonstrated resolution of the contrast extravasation into the hematoma cavity, with no significant change in its overall size. However, AF recurred, likely due to postablation inflammation or increased left atrial pressure from the hematoma. Anticoagulation was resumed with intravenous heparin instead of rivaroxaban, as heparin could be promptly reversed in the event of the hematoma expansion. Low-flow supplemental oxygen was required owing to pulmonary edema secondary to elevated left atrial pressure, but the patient's vital signs remained stable. His only symptom was mild chest discomfort during inspiration. Serial TTE showed no significant change in hematoma size, although gradual decrease in its echogenicity was observed. On postoperative day 4, however, a sudden reduction in hematoma size was observed (Figure 5A). Cardiac CT confirmed on axial view that the hematoma decreased to 52 × 14 mm (Figure 5B). Because of concerns about possible rupture or embolization, a contrast-enhanced whole-body CT was performed, which demonstrated no evidence of embolism. No neurological deficits or other symptoms suggesting embolism were observed. Subsequently, a slight re-expansion occurred, followed by a sustained gradual reduction in hematoma size (Figures 6A and 6B). After rivaroxaban 15 mg was resumed on postoperative day 8, there were no further changes in hematoma size or hemodynamic status. The patient remained clinically stable and was discharged on postoperative day 13.

## Outcome and Follow-Up

In the outpatient setting, follow-up TTE was performed monthly. Serial examinations showed continued regression of the hematoma, and at the 4-month follow-up, TTE revealed near-complete resolution (Figures 6C and 6D). No recurrence of AF was observed during the follow-up period.

## Discussion

LAIH, defined as a false, blood-filled cavity within the left atrial wall or interatrial septum, is an extremely rare complication of catheter ablation. More than half of the reported cases are related to mitral valve surgery, with catheter ablation accounting for only about 8% of cases.^1^ Other etiologies include percutaneous coronary intervention, aortic valve surgery, myocardial infarction, and coronary artery bypass grafting.^1^ The true incidence of LAIH remains unknown, as most reports are individual case studies. Nevertheless, the number of reported cases has increased with advances in echocardiographic detection and the rising frequency of left atrial catheter interventions.^1^^,^^2^ In addition, the expanding use of large-bore sheaths in procedures such as transcatheter edge-to-edge repair and left atrial appendage occlusion may further elevate the relative risk of LAIH.^3^^,^^4^

**Figure 7:**
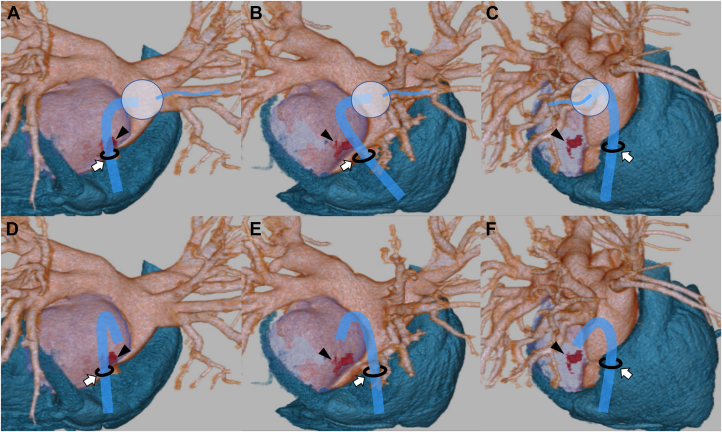
Ideal RIPV Cannulation and the Anatomical Relationship Between Contrast Extravasation Site and Sheath Trajectory (A to C) Ideal cannulation to the inferior branch of the RIPV. (D to F) Anatomical relationship between the site of contrast extravasation within the hematoma and the sheath trajectory. The inferomedial extravasation site anatomically aligns with the sheath trajectory during inferior branch cannulation of the RIPV. (A and D) Posterior view, (B and E) right-posterior oblique view, and (C and F) right view. The white arrow denotes the Brockenbrough puncture point, and the black arrowhead denotes the contrast extravasation site. RIPV = right inferior pulmonary vein.

The possible mechanisms of LAIH include interatrial transseptal puncture, forceful contact with mapping or ring catheters, and blunt trauma with a large-bore sheath.^5^, ^6^, ^7^ In the present case, the left atrium was not dilated, and a 15-F large-bore sheath was markedly deflected while reselecting the inferior branch of the RIPV. Furthermore, contrast extravasation into the hematoma was detected in the inferomedial region. The inferomedial extravasation site anatomically aligns with the sheath trajectory during inferior branch cannulation of the RIPV, supporting sheath-related blunt injury as the mechanism (Figure 7). Another possible mechanism of blunt injury was that, after the Brockenbrough puncture, the large-bore sheath was advanced into the left atrium and inadvertently impinged on the posterior wall before being properly deflected. A relatively small left atrial size may have been a potential risk factor for these events.

Three major clinical concerns were relevant in this case. First, pulmonary edema due to pulmonary venous outflow obstruction could have caused respiratory compromise. Although no significant compression or obstruction of the pulmonary veins was noted on cardiac CT, progressive elevation of left atrial pressure due to hematoma expansion remained a potential risk. Fortunately, the patient's respiratory condition remained stable, with only low-flow oxygen therapy initiated. This stability may have been aided by left-to-right shunting across the iatrogenic atrial septal defect created during transseptal puncture, which likely mitigated the elevation in left atrial pressure. Second, there was a risk of obstruction or entrapment of the mitral valve inflow by the hematoma. Because narrowing of the left ventricular inflow tract was observed, intravenous fluid was administered to maintain adequate left ventricular preload. As entrapment of the mitral valve could result in hemodynamic collapse, venoarterial extracorporeal membrane oxygenation was kept on standby. Third, there was concern that hematoma expansion might lead to cardiac tamponade or myocardial rupture. However, contrast extravasation into the hematoma disappeared on follow-up CT the next day, and the hematoma remained stable in size without pericardial effusion.

Regarding therapeutic strategies, surgical evacuation is generally recommended in hemodynamically unstable patients.^6^^,^^8^ Conversely, in hemodynamically stable patients, conservative management is often appropriate, as spontaneous resolution is frequently observed.^7^^,^^9^ In the present case, early detection by routine intraoperative intracardiac echocardiography allowed prompt intervention—including protamine reversal of heparin, strict bed rest, and close monitoring—thereby preventing hematoma expansion and secondary complications.

The hematoma gradually decreased in size, with reduced echogenicity on serial echocardiography, likely reflecting progressive liquefaction and resorption. Although a sudden reduction in size raised concern for rupture or embolization, no evidence of systemic embolism or neurological deficits was observed. Anticoagulation therapy was cautiously resumed, and the patient experienced a favorable clinical course.

## Conclusions

LAIH is a rare but serious complication of catheter ablation. Careful sheath manipulation and vigilant echocardiographic monitoring are essential for prevention and early detection. In hemodynamically unstable patients, surgical intervention is often unavoidable. However, in hemodynamically stable patients, early recognition and timely intervention can enable successful conservative management, leading to complete resolution.

## Funding Support and Author Disclosures

The authors have reported that they have no relationships relevant to the contents of this paper to disclose.

**Take-Home Messages**

• Left atrial intramural hematoma can be effectively managed with conservative therapy in hemodynamically stable patients.
• Early recognition and timely intervention are crucial for achieving complete resolution.

**Equipment List:** 

Imaging • Intracardiac echocardiography (ViewFlex Xtra ICE catheter, Abbott Medical Japan)
Access • SLO 8.5-F and 0.032 angled wire (Abbott Medical Japan) SLO l0-F (Abbott Medical Japan) • Supersheath II 6-F 25 cm (Medikit) • Cryoballoon sheath (FlexCath Advance 15-F, Medtronic)
Interatrial transseptal puncture • Scooper RFN standard (Fukuda Denshi) • SLO 8.5-F and 0.032 angled wire (Abbott Medical Japan) • GuideRight (0.032 wire) (Abbott Medical Japan)
Ablation • Cryoballoon (Arctic Front Advance Pro 28 mm, Medtronic) • Mapping system (EnSite Precision system, Abbott Medical Japan) • Ring catheter (Inquiry Optima l, l-20 mm, Abbott Medical Japan) • Intracardiac electrode catheter (Scooper 5-F large C curve, Fukuda Denshi) • Mapping catheter (Achieve Advance 20 mm, Abbott Medical Japan) • Esophageal temperature probe (Fe-po ET-Watcher, Fukuda Denshi)
